# Predictors for the development of motoric cognitive risk syndrome in older adults

**DOI:** 10.1186/s12877-024-05179-8

**Published:** 2024-07-03

**Authors:** Nurul Fatin Malek Rivan, Arimi Fitri Mat Ludin, Brian C. Clark, Suzana Shahar

**Affiliations:** 1 Nutritional Sciences Programme and Centre for Healthy Ageing and Wellness (H-CARE), Faculty of Health Sciences, Universiti Kebangsaan Malaysia, Kuala Lumpur, Malaysia; 2 Biomedical Science Programme and Centre for Healthy Ageing and Wellness (H-CARE), Faculty of Health Sciences, Universiti Kebangsaan Malaysia, Jalan Raja Muda Abdul Aziz, Kuala Lumpur, 50300 Malaysia; 3 Ohio Musculoskeletal and Neurological Institute (OMNI) and Department of Biomedical Sciences, Ohio University, Athens, OH USA; 4 Dietetics Programme and Centre for Healthy Ageing and Wellness (H-CARE), Faculty of Health Sciences, Universiti Kebangsaan Malaysia, Kuala Lumpur, Malaysia

**Keywords:** MCR, Gait impairments, Subjective cognitive decline, Incidence, Predictors, Older adults

## Abstract

**Background:**

Motoric cognitive risk (MCR) syndrome refers to a condition where both slow gait and memory complaints coexist, which heightens their vulnerability to developing dementia. Considering that the risk factors of MCR are elucidated from cross-sectional studies and also likely vary based on socioeconomic status, we conducted a community-based longitudinal study to determine the predictors of MCR among older adults in Malaysia.

**Methods:**

Out of 1,249 older participants (aged 60 years and above) without MCR at baseline (Wave II of LRGS-TUA cohort study), 719 were successfully followed up after 3.5 years to identify predictors of subsequent MCR development. A comprehensive interview-based questionnaire was administered for sociodemographic information, cognitive function, psychosocial, functional status, and dietary intake. Anthropometric measurements, body composition, and physical performance were assessed. Univariate analyses were performed for each variable, followed by a hierarchical logistic regression analysis to identify the predictors of MCR that accounted for confounding effects between the studied factors.

**Results:**

The incidence rate of MCR was 4.0 per 100 person-years. Smoking (Adjusted Odd Ratio (Adj OR) = 1.782; 95% Confidence Interval (CI):1.050–3.024), hypertension (Adj OR = 1.725; 95% CI:1.094–2.721), decreased verbal memory as assessed by the lower Rey Auditory Verbal Learning Test (RAVLT) (Adj OR = 1.891; 95% CI:1.103–3.243), and decreased functional status measured using instrumental activity of daily living (IADL) (Adj OR = 4.710; 95% CI:1.319–16.823), were predictors for MCR incidence.

**Conclusions:**

Our study results provide an initial reference for future studies to formulate effective preventive management and intervention strategies to reduce the growing burden of adverse health outcomes, particularly among Asian older adults.

## Introduction

The accelerated ageing population is expected to increase age-associated pathological conditions, such as gradual loss of health and physical strength, making older adults vulnerable to adverse events such as disability, dependency, falls, or even mortality [1, 2]. Past studies have indicated that the coexistence of reduced gait speed with cognitive complaints to be a common condition in older adults and may increase the risk of dementia [1, 3]. Thus, motoric cognitive risk (MCR) syndrome was introduced as a predementia syndrome characterised by the simultaneous presence of subjective cognitive complaints and slow gait in older adults without dementia or mobility disability [1]. MCR can be detected without time-consuming comprehensive cognitive tests or other burdensome investigations, enhancing its accessibility in various clinical settings [4].

Besides that, frailty is a common geriatric syndrome characterised by an age-related decline in physiologic reserves and function of multiple systems and often co-exist with cognitive impairment [5]. Thus, the construct of cognitive frailty was introduced by incorporating physical frailty and mild cognitive impairment (MCI) without an overt dementia diagnosis [6]. Cognitive frailty is also known as a pre-dementia state [7] with comprehensive screening tools to identify those older adults with cognitive frailty. The fact that subjective cognitive complaints predict MCI incidence and dementia [8] suggests that MCR might represent an earlier stage of preclinical dementia and cognitive frailty. A cross-sectional study indicated that the older adults with MCR syndrome had the highest prevalence of frailty, pain, depression, functional limitation, and lowest perceived health, with the most remarkable improvement in cognition after three months of a dual-task exercise intervention [9]. Thus, an effort to interventionally target older adults with MCR should be a public health priority to delay the onset of cognitive frailty and adverse health outcomes among older adults.

Prior work has reported that the risk factors such as diabetes, depressive symptoms, falls, and obesity were associated with increased risk of MCR syndrome [10]. Cardiovascular diseases and cardiovascular risk factors, such as hypertension and diabetes, are often reported to increase the MCR risk, similar to dementia and mild cognitive impairment (MCI) [11, 12]. Other significant risk factors for MCR examined in individual studies were arthritis, poor vision, and living in rural areas [4, 12]. However, all of these studies were conducted cross-sectionally. There are several cohort studies involving MCR analysis; however, these studies were conducted to identify its predicting ability on age-related negative health outcomes [13, 14].

To our knowledge, no study has identified the predictors of MCR involving a broad aspect of assessments through a longitudinal study. It is noteworthy that the prevalence analysis only provides a snapshot of the condition of a population at a particular point in time and is unable to explain a cause-and-effect relationship between the studied variables. Besides, the targeted older adults involved in this study, which were multi-ethnic Asian older adults, also came from low educational and socioeconomic status, where the factors contributing to the MCR development might differ from prior works. Individuals with limited education and financial resources may face challenges accessing healthcare, engaging in cognitively stimulating activities, and adopting healthy lifestyle behaviours. Factors such as diet, physical activity levels, social support networks, and access to healthcare services can vary significantly within this demographic group and may impact cognitive and motor function differently compared to other populations. Thus, this prospective cohort study aimed to identify the predictors of MCR and to estimate the strength of the combined factors in identifying MCR risk among Malaysian older adults during a 3.5-year follow-up.

## Methods

### Study design and participants

This is a follow-up study of the Longitudinal Study on Neuroprotective Model for Healthy Longevity (LRGS-TUA) cohort at five years endpoint [15]. The secondary data of Wave II (18-month) from the LRGS-TUA database were analysed as a baseline for this study. Notably, the prevalence analysis of this phase has been reported by previous findings [12]. This study was conducted in four states of Malaysia (Selangor, Perak, Kelantan, and Johor), representing central, north, east and south zone from November 2014 till September 2015. Inclusion criteria were individuals aged 60 years and above, who did not have psychiatric and mental disorders (including dementia), no terminal illnesses, and preserved functional ability. Those participants with the Malay-Mini Mental State Examination (M-MMSE) score of 14 and below were excluded because this cut-off indicated moderately severe or severe cognitive impairment. Excluding individuals with such impairment ensured that the study focused on participants with preserved cognitive function, as MCR is considered a predementia syndrome characterized by subtle cognitive decline and motor impairments.

**Fig. 1:**
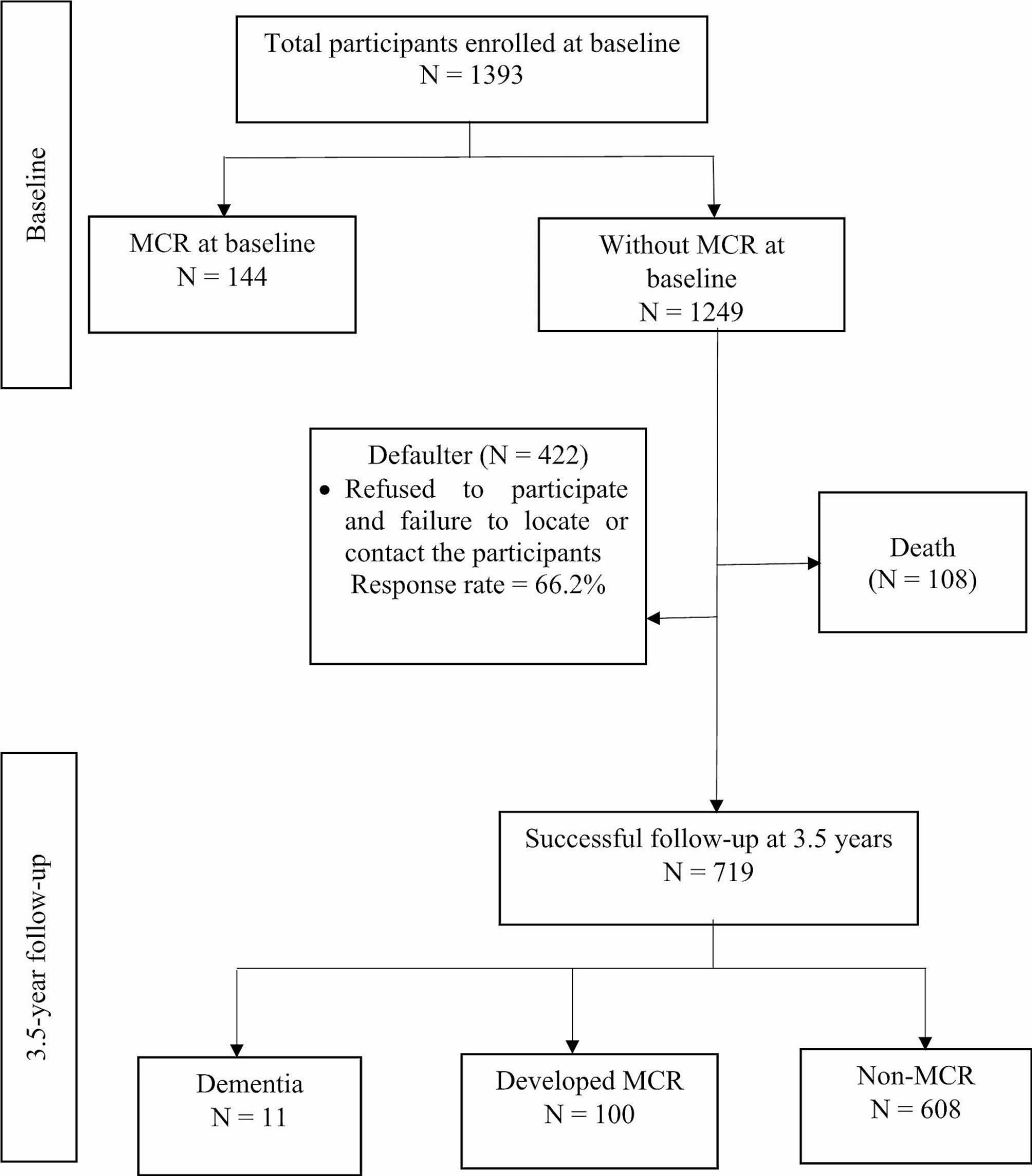
Illustration of the number of participants from the baseline to the 3.5-year follow-up for MCR. *Notes* MCR = Motoric cognitive risk

At baseline, a total of 1,393 participants were recruited through multi-stage random sampling procedures. Specified areas were chosen for the study if the population of these locations comprised at least 10% of older adults [15]. Figure 1 shows that 144 participants were categorised as having MCR syndrome at baseline, while another 1,249 participants were grouped in the Non-MCR syndrome group at baseline. It is noteworthy that only Non-MCR participants at baseline were included in the follow-up assessments for the incidence analysis. The incidence of MCR referred to MCR development during the 3.5-year follow up. After 3.5 years, 108 participants died, 422 failed to be located or contacted, and some refused or had no transportation to participate in the follow-up assessments at the community centres. Hence, a total of 719 Non-MCR participants at baseline were successfully followed up at 3.5 years. Different fieldworkers were recruited for the follow-up assessments to comply with the blinded assessors’ criteria. This study was approved by the Medical Research and Ethics Committee of the Universiti Kebangsaan Malaysia (UKM1.21.3/244/NN-2018-145). Written information was provided, and informed written consent was obtained from all participants before participation.

### Data collection

Participants underwent interviews by trained enumerators who utilised a structured questionnaire and measurements for a number of parameters at their respective community centres. The questionnaire consists of information on socio-demography, medical history, anthropometry, blood pressure, body composition, cognitive and physical assessments, psychosocial and functional status, and dietary intake as reported by Shahar et al. [15]. The descriptions of the measuring instruments are detailed below. The participants were allowed to rest between the tests, and monetary incentives were provided for those who completed the assessment. Because these factors could depend on an individual’s cognitive status, participants were stratified at baseline based on the MCR status.

### Incidence of MCR

MCR incidence referred to MCR development during the 3.5-year follow-up of the participants who did not exhibit MCR at the baseline. MCR syndrome was first proposed by Verghese et al. [1], which is a high-risk clinical syndrome with strong predictive validity for dementia. Participants were defined as having MCR syndrome if they satisfied the operationalisation definition of MCR proposed by the previous report [12].

Participants were defined as not having moderately severe or severe dementia if they scored 15 and above in M-MMSE. The MMSE score criteria used in this study are based on established guidelines [16]. A score of 15 or higher was selected as the threshold for not having dementia, as scores below this level generally indicate moderately severe to severe cognitive impairment, which strongly associated with dementia. This cutoff was chosen to ensure a balanced assessment of cognitive function in our study population, as our participants had lower educational levels and low literacy rates, considering both sensitivity (35.4%) and specificity (76.8%) for dementia detection [17]. A single dichotomous question, “Do you feel you have more problems with memory than most?” on the Geriatric Depression Scale (GDS), was asked by trained enumerators to elicit the presence of subjective memory complaints. Participants who answered “yes” on this question were defined as having subjective memory complaints. Moreover, preserved activities of daily living, including eating/feeding, dressing, bathing and showering, functional mobility, climbing up and down the stairs, personal hygiene and grooming, and toilet hygiene, were assessed using the activities of daily living (ADL) questionnaire [18]. Gait speed was measured using a 6 m walk on a level floor over time. Participants were instructed to walk back and forth over the marked distance at their usual pace. Slow gait was defined as one standard deviation (SD) below mean population gait speed (Cut-off point: 0.71 m/s) [1]. Thus, participants who fulfilled these criteria were categorised as MCR group while the remaining participants were grouped as Non-MCR. Meanwhile, participants with severe cognitive impairments (M-MMSE *≤* 14) were excluded from the analysis.

### Possible predictors of MCR

As previously stated, the protocol of the baseline measurements has been reported in previously published literature [15].

#### i. Sociodemographic information and medical status

The sociodemographic information was obtained, including age, gender, ethnicity, strata, years of formal education, marital status, living arrangement, household income, and smoking status. The self-reported medical history on certain diseases (diabetes mellitus, hypertension, hypercholesterolemia, stroke, arthritis, heart diseases and cancer) diagnosed by doctors in the prior years were recorded.

#### ii. Anthropometry, blood pressure and body composition

Anthropometry measurements including weight, height, waist circumference, hip circumference, mid-upper arm circumference (MUAC), and calf circumference were recorded. The body mass index (BMI, kg/m^2^) was calculated as the weight in kilograms divided by the squared standing height in meters. All the circumferences were measured using a non-extensible and flexible plastic measuring tape and were done according to standard protocols [15]. The body composition was assessed via Bio-electrical Impedance Analysis (BIA) Inbody S10 (Bio-space Co. Ltd, Seoul, Korea). The systolic and diastolic blood pressure was assessed using a calibrated digital automatic blood pressure monitor (OMRON, Kyoto, Japan).

#### iii. Cognitive assessments

Global cognitive function was assessed using the Malay version of the Montreal Cognitive Assessment (MoCA) [19]; the attention and working memory were evaluated using the Weschler Memory Scale-Revised (WMS-R) digit span [20]; information processing speed was measured using the digit symbol test; verbal learning and memory were assessed using the Rey Auditory Verbal Learning Test (RAVLT) [21].

#### iv. Physical assessments

Physical assessments were done using the senior fitness test (SFT) [22]. A total of five fitness tests were conducted, including a 2-minute step test (endurance), back scratch test (upper body flexibility), chair sit and reach test (lower body flexibility), chair stand test (lower body strength), timed-up and go-test (TUG) (balance and mobility), and handgrip strength (upper body strength).

#### v. Psychosocial and functional status

The validated Malay version GDS (GDS-15) was used to assess potential depressive symptoms among the participants [23], while the functional status was evaluated using the instrumental activity of daily living (IADL) [24].

#### vi. Dietary intake

The dietary intake was obtained using the dietary history questionnaire (DHQ) [25]. The nutrient intake was further analysed using the Nutritionist Pro software. The output from the software was then exported into the Excel database.

### Statistical analysis

The cumulative incidence of MCR was determined by dividing the number of new MCR cases observed during the follow-up period by the number of at-risk participants in the population at baseline [26]. The incidence rates of MCR were obtained by dividing the number of new MCR cases by the total person-time detected between the two assessments [26]. The number of at-risk participants and total person-time referred to participants with no MCR specifically at baseline.

Descriptive statistics were used to present the baseline characteristics of the participants based on the MCR status at 3.5 years. The dependent variable for the predictor’s analysis was the MCR group as the reference variable, compared to the non-MCR group. All the potential predictors of MCR were compared between MCR and Non-MCR using a chi-square test (X^2^) for categorical variables and an independent t-test for continuous variables. The results were reported as n (%) and the mean ± standard deviation for normally distributed data. The significant value was set at *p* < 0.05.

As conducted by previous local studies [27, 28], a hierarchical binary logistic regression (BLR) was performed to determine MCR predictors in a multivariate model. For the first stage, all the significant variables (*p* < 0.05) from the univariate analysis were adjusted for multiple testing and classified into five different groups, as follows: [1] sociodemographic and medical status; [2] blood pressure, anthropometry, and body composition; [3] cognitive and physical assessments; [4] psychosocial and functional status; and [5] dietary intake associated with cognitive frailty. Then, all significant variables (*p* < 0.05) from each model were included in the final logistic model. The significant variables that appeared in the final model were the predictors of MCR among participants (*p* < 0.05) and entered into the model for predicting MCR risk. Scores for the remaining variables in the logistic regressions were calculated to an integer score based on the odds ratios (ORs). Finally, receiver operating characteristic curve (ROC) analysis has been conducted to identify the best cut-off score for the MCR predictors, with maximum sensitivity and specificity. The area under the curve (AUC) from ROC analysis was compared to identify the model that had a better association with MCR risk. These analyses were performed using SPSS version 23.0 (Licensed materials - Property of SPSS Incorporation an IBM Company Copyright 1989 and 2010 SPSS, Chicago, United States).

## Results

### Incidence of MCR syndrome

In this study, out of 1,249 participants without MCR at baseline, a total of 719 participants were successfully followed up (Response rate: 66.2%). In the present study, 608 participants (84.6%) remained without MCR, 100 (13.9%) developed MCR, while 11 (1.6%) of them developed dementia after 3.5 years. Those participants diagnosed with dementia (*n* = 11) were excluded from the analysis to focus more precisely on identifying factors that contribute to the early stages of cognitive decline and motor impairment. In this group, the cumulative incidence of MCR was 13.9%. The observed incidence rate of MCR within the 3.5 years had a value of 4.0 per 100 person-years.

### Potential predictors of MCR

As stated in Table 1, those in the MCR group (76.34 ± 5.93 years old) were significantly older than participants who did not have MCR (75.10 ± 4.86 years old) (*p* < 0.05). The MCR group (4.73 ± 3.90 years) had significantly lower years of formal education than the non-MCR group (5.87 ± 3.93 years) (*p* < 0.05). Generally, the MCR participants (59.0%) had a higher prevalence of hypertension than those in the non-MCR group (41.0%) (*p* < 0.05).

Moreover, a lower body fat percentage was observed among MCR participants (35.87 ± 9.48%) than the participants in the non-MCR group (38.25 ± 9.32%) (*p* < 0.05). The diastolic blood pressure (DBP) was lower among the MCR group (72.40 ± 11.75 mmHg) than the Non-MCR group (75.11 ± 11.89 mmHg) (*p* < 0.05). The functional status of the participants, as indicated by the IADL score, was lower among the MCR group (12.89 ± 2.13) compared to the Non-MCR group (13.29 ± 1.55) (*p* < 0.05). Besides that, the MCR group showed a lower performance in the RAVLT test of Trial 1–5 (23.14 ± 11.61) compared to those in the Non-MCR group (28.34 ± 13.14)(*p* < 0.05).

There were no group differences in most of the physical fitness test, including TUG test, hand grip test, chair stand test and back scratch test (*p* > 0.05), with an exception of chair sit and reach test. The MCR group (3.99 ± 10.68 cm) had lower physical performance, as indicated by the chair sit and reach test, than the Non-MCR group (1.58 ± 10.44 cm) (*p* < 0.05). As shown in Table 2, the dietary vitamin E intake also was lower among the MCR group (11.64 ± 36.70 mg/day) than the Non-MCR group (27.91 ± 23.99 mg/day) (*p* < 0.05).

**Table 1:** The baseline attributes of the participants with MCR and Non-MCR at 3.5-years follow-up [presented as mean ± standard deviation (sd) or n (%)]

Baseline Parameters	Total (*N* = 708)	Non-MCR (*N* = 608)	MCR (*N* = 100)	*p*-Value
Age, mean ± sd:	75.28 ± 5.04	75.10 ± 4.86	76.34 ± 5.93	0.023*
Gender:				
Men	354 (50.0)	296 (48.7)	58 (58.0)	0.105
Women	354 (50.0)	312 (51.3)	42 (42.0)	
Ethnicity				
Malay	437 (61.7)	382 (62.8)	55 (55.0)	0.066
Chinese & Indian	271 (38.3)	226 (37.2)	45 (45.0)	
Strata:				
Urban	381 (53.8)	331 (54.4)	50 (50.0)	0.449
Rural	327 (46.2)	277 (45.6)	50 (50.0)	
Education (years), mean (sd):	5.30 ± 3.92	5.87 ± 3.93	4.73 ± 3.90	0.007**
Married	526 (74.3)	450 (74.0)	76 (76.0)	0.713
Living alone	64 (9.0)	54 (8.9)	10 (10.0)	0.707
Smoker	118 (16.7)	96 (15.8)	22 (22.0)	0.146
Household income, mean ± sd (Ringgit Malaysia, RM)	1391.44 ± 2469.99	1425.03 ± 2609.04	1186.12 ± 1326.87	0.375
Diseases:				
Diabetes mellitus	170 (24.0)	140 (23.0)	30 (30.0)	0.131
Hypertension	345 (48.7)	286 (47.0)	59 (59.0)	0.031*
Hypercholesterolemia	290 (41.0)	241 (39.6)	49 (49.0)	0.080
Stroke	9 (1.3)	7 (1.2)	2 (2.0)	0.370
Arthritis	174 (24.6)	147 (24.2)	27 (27.0)	0.533
Heart Disease	54 (7.6)	44 (7.2)	10 (10.0)	0.314
Cancer	8 (1.1)	7 (1.2)	1 (1.0)	1.000
BMI (kg/m^2^), mean ± sd	25.24 ± 4.16	25.33 ± 4.24	24.67 ± 3.62	0.145
BMI, WHO category				
Underweight	22 (3.1)	20 (3.3)	2 (2.0)	0.572
Normal	331 (46.8)	279 (45.9)	52 (52.0)	
Overweight	271 (38.3)	234 (38.5)	37 (37.0)	
Obesity	84 (11.9)	75 (12.3)	9 (9.0)	
Calf Circumference (cm), mean ± sd	33.46 ± 3.61	33.50 ± 3.66	33.20 ± 3.30	0.439
Waist Circumference (cm), mean ± sd	82.49 ± 11.53	82.34 ± 11.71	83.43 ± 10.33	0.381
Hip Circumference (cm), mean ± sd	93.80 ± 9.42	93.86 ± 9.56	93.40 ± 8.55	0.653
MUAC (cm), mean ± sd	27.58 ± 3.17	27.61 ± 3.19	27.44 ± 3.19	0.620
Body composition:				
% Body Fat, mean ± sd	37.92 ± 9.38	38.25 ± 9.32	35.87 ± 9.48	0.019*
Skeletal muscle mass (kg), mean ± sd	19.35 ± 4.38	19.23 ± 4.41	20.05 ± 4.15	0.085
Blood Pressure:				
Systolic (mmHg), mean ± sd	136.71 ± 21.19	136.59 ± 21.45	137.51 ± 19.58	0.691
Diastolic (mmHg), mean ± sd	74.73 ± 11.90	75.11 ± 11.89	72.40 ± 11.75	0.036*
Psychosocial and functional status:				
GDS, mean ± sd	2.77 ± 2.17	2.75 ± 2.16	2.91 ± 2.28	0.499
IADL, mean ± sd	13.23 ± 1.65	13.29 ± 1.55	12.89 ± 2.13	0.024*
Neurocognitive test:				
MoCA, mean ± sd	19.79 ± 5.18	19.97 ± 5.14	18.65 ± 5.32	0.018*
Span Digit, mean ± sd	7.94 ± 2.45	7.96 ± 2.41	7.88 ± 2.69	0.775
RAVLT Trial 1–5, mean ± sd	24.43 ± 13.95	28.34 ± 13.14	23.14 ± 11.61	< 0.001***
Digit symbol, mean ± sd	5.59 ± 3.29	5.65 ± 3.31	5.22 ± 3.17	0.226
VR I, mean ± sd	40.51 ± 33.54	40.85 ± 33.57	38.45 ± 33.42	0.508
VR II, mean ± sd	42.19 ± 35.95	42.62 ± 35.96	39.56 ± 35.94	0.431
Fitness test				
2-minute step test (no of steps), mean ± sd	63.24 ± 21.92	63.73 ± 21.62	60.16 ± 23.60	0.140
Hand grip (kg), mean ± sd	23.81 ± 7.56	23.62 ± 7.52	24.95 ± 7.69	0.104
Chair stand test (no of complete chair stand), mean ± sd	10.45 ± 2.74	10.46 ± 2.68	10.41 ± 3.04	0.887
Chair sit and reach test (cm), mean ± sd	1.92 ± 10.50	1.58 ± 10.44	3.99 ± 10.68	0.034*
TUG test (sec), mean ± sd	10.56 ± 2.33	10.56 ± 2.19	10.61 ± 3.07	0.823
Back scratch test (cm), mean ± sd	14.82 ± 11.29	14.75 ± 11.35	15.30 ± 10.95	0.656

* Significant at *p* < 0.05. *Notes* MCR = Motoric cognitive risk; BMI = Body mass index; MUAC = Mid-upper arm circumferences; ADL = activities of daily living; IADL = instrumental activities of daily living; GDS = Geriatric Depression Scale; MMSE = Mini-Mental State Examination; MoCA = Montreal Cognitive Assessments; RAVLT = Rey Auditory Verbal Learning Test; VR I = visual reproduction I; VR II = visual reproduction II; TUG = Timed-up-and-Go test; and sd = standard deviation; WHO = World Health Organization

**Table 2:** The dietary intake among participants at the baseline with MCR and Non-MCR at the 3.5-years follow-up [presented as mean ± standard deviation (sd) or n (%)]

Baseline Parameters	Total (*N* = 708)	Non-MCR (*N* = 608)	MCR (*N* = 100)	*p*-Value
Energy (kcal)	1557 ± 424	1549 ± 423	1606 ± 431	0.267
Protein (g/day)	66.19 ± 21.05	66.39 ± 21.29	64.86 ± 19.51	0.517
Carbohydrate (g/day)	210.93 ± 64.19	212.38 ± 64.53	201.69 ± 61.49	0.138
Fat (g/day)	55.49 ± 19.92	55.91 ± 20.36	52.85 ± 16.73	0.170
Cholesterol (mg/day)	258.53 ± 206.78	258.14 ± 211.69	261.02 ± 173.31	0.901
SFA (g/day)	7.29 ± 4.20	7.32 ± 4.23	7.10 ± 4.00	0.646
MUFA (g/day)	6.21 ± 5.95	6.23 ± 3.26	6.04 ± 3.10	0.596
PUFA (g/day)	4.43 ± 2.77	4.46 ± 2.83	4.25 ± 2.37	0.485
Total Fibre (g/day)	5.14 ± 3.12	5.15 ± 3.13	5.07 ± 3.11	0.824
Vitamin A (RE/day)	758.58 ± 489.31	759.85 ± 508.42	750.54 ± 346.20	0.865
Vitamin C (mg/day)	95.50 ± 72.93	96.86 ± 75.72	86.91 ± 51.37	0.224
Vitamin D (mg/day)	0.18 ± 2.67	0.20 ± 2.87	0.07 ± 0.24	0.666
Vitamin E (mg/day)	25.69 ± 16.15	27.91 ± 23.99	11.64 ± 36.70	0.011*
Vitamin K (mg/day)	20.11 ± 50.29	20.67 ± 52.60	16.57 ± 31.94	0.467
Alpha-Tocopherol (mg/day)	0.04 ± 0.22	0.04 ± 0.22	0.05 ± 0.25	0.530
Thiamin (mg/day)	0.77 ± 0.35	0.78 ± 0.35	0.72 ± 0.35	0.158
Riboflavin (mg/day)	1.08 ± 0.41	1.09 ± 0.40	1.03 ± 0.42	0.207
Niacin (mg/day)	10.33 ± 4.05	10.38 ± 4.17	10.04 ± 3.19	0.448
Pyridoxine (mg/day)	0.93 ± 0.50	0.93 ± 0.50	0.95 ± 0.51	0.778
Panthotenic acid (mg/day)	0.13 ± 0.40	0.13 ± 0.39	0.14 ± 0.48	0.855
Folate (µg /day)	108.91 ± 74.06	110.44 ± 76.81	99.21 ± 52.65	0.177
Calcium (mg/day)	448.35 ± 245.79	450.94 ± 254.91	431.91 ± 177.51	0.490
Sodium (mg/day)	3067.89 ± 1297.67	3056.79 ± 1286.69	3138.32 ± 1370.56	0.576
Potassium (mg/day)	1474.87 ± 513.60	1477.04 ± 519.76	1461.06 ± 475.05	0.782
Iron (mg/day)	14.94 ± 8.16	15.04 ± 8.40	14.33 ± 6.40	0.438
Zinc (mg/day)	3.81 ± 2.20	3.84 ± 2.27	3.57 ± 1.63	0.267
Copper (mg/day)	0.51 ± 0.40	0.52 ± 0.40	0.46 ± 0.41	0.229
Magnesium (mg/day)	126.33 ± 56.90	126.59 ± 57.59	124.71 ± 52.59	0.769
Manganese (mg/day)	0.58 ± 0.73	0.60 ± 0.75	0.50 ± 0.64	0.244

* Significant at *p* < 0.05 using Independent t-test; *Notes* sd = standard deviation

### Predictors of MCR syndrome

In Table 3, the results from the hierarchical binary logistic regression (BLR) indicated that smoking (Adjusted Odd Ratio (Adj OR) = 1.782; 95% Confidence Interval (CI): 1.050, 3.024; *p* < 0.05), hypertension (Adj OR = 1.725; 95% CI: 1.094, 2.721; *p* < 0.05), low performance in RAVLT test (i.e., decreased verbal memory) (Adj OR = 1.891; 95% CI: 1.103, 3.243; *p* < 0.05), and low functional status as indicated by a lower score in IADL (Adj OR = 4.710; 95% CI: 1.319, 16.823; *p* < 0.05) were found to be a predictor of MCR in this study by controlling the gender, marital status, strata and living arrangement. However, age, years of education, body fat percentage, and vitamin E intake were no longer associated with the MCR incidence in the multivariate analysis (*p* > 0.05).

**Table 3:** Potential predictors for MCR at 3.5 years (*N* = 708)

Predictor of interest	B	SE	Adj OR (95% CI)	*p*-Value
Age (years)	0.031	0.022	1.032 (0.988–1.077)	0.156
Education	0.076	0.266	1.079 (0.640–1.817)	0.776
* ≤* 6 years				
> 6 years (ref)				
Smoking status	0.578	0.270	1.782 (1.050–3.024)	0.032*
Yes				
No (ref)				
Hypertension	0.545	0.232	1.725 (1.094–2.721)	0.019*
Yes				
No (ref)				
Body fat (%)	0.441	0.343	1.554 (0.794–3.041)	0.198
High				
Normal (ref)				
RAVLT Trial 1–5	0.637	0.275	1.891 (1.103–3.243)	0.021*
Low				
High (ref)				
IADL	1.550	0.650	4.710 (1.319–16.823)	0.017*
Dependent				
Independent (ref)				
Vitamin E (mg/day)	0.159	0.311	1.172 (0.637–2.158)	0.610
Low				
Normal (ref)				

* Significant at *p* < 0.05 using Hierarchical Binary Logistic Regression. *Notes* SE = Standard error; Adj OR = Adjusted odd ratio; IADL = Instrumental activities of daily living; RAVLT = Rey Auditory Verbal Learning Test

To facilitate the use of the present model in a community or in clinical practice for MCR risk identification, the OR scores were rounded to the nearest integer, as shown in Table 4. The total score ranged from zero, without any risk factor, to 10, with all the risk factors present. The ROC curves with an area under the curve (AUC) score of 0.636 indicates an acceptable range for screening older adults with a high risk of MCR. The best cut-off of the total Model 2 to identify older people with a high risk of MCR was *≥* 4, with 90% specificity.

**Table 4:** Predictor coefficients for MCR predicting model

Model	B	SE	Adj OR (95% CI)	Points
Smoking status	0.578	0.270	1.782 (1.050–3.024)	2
Hypertension	0.545	0.232	1.725 (1.094–2.721)	2
RAVLT Trial 1–5	0.637	0.275	1.891 (1.103–3.243)	2
IADL status	1.550	0.650	4.710 (1.319–16.823)	4
**AUC**	**0.636**	**0.030**	**0.568–0.684**	

*Abbreviations* B = regression coefficient; SE = Standard error; Adj OR = Adjusted odd ratio; IADL = Instrumental activities of daily living; AUC = Area under the curve; RAVLT = Rey Auditory Verbal Learning Test. Notes: Points are rounded numbers of odds ratio scores for predicting the MCR predicting model

## Discussion

In a prior study, Lau et al. [12] reported that women, living in rural areas, had obesity, diabetes, heart disease, and cancer are risk factors for MCR using LRGS TUA data at 18-month of follow-up. However, all of these risk factors were diminished when assessed longitudinally, likely indicating that these prior identified factors did not cause an effect on the occurrence of MCR among our local population per se. A possible explanation could be that the previous study only included low-income older adults (household income of less than RM3900/USD 819 per month), while this study was a random sampling of all eligible older participants in the catchment areas. Thus, it is plausible to observe a significant association between women and those living in rural areas with MCR, as these populations were known to have a low-income status [29, 30]. Besides that, this study did not identify obesity, diabetes, heart disease, and/or cancer as a predictor of MCR incidence, suggesting that the development of MCR may not be caused by the mechanisms that are shared with these non-longitudinal factors. For instance, the population involved in this study had a lower prevalence of the reported morbidities than the prior works [12], which could limit the statistical evidence to detect the associations between these factors and MCR incidence.

This current study revealed that older adults who smoke had a higher risk of developing MCR syndrome. Smoking is well known to contribute to an increased risk of arterial diseases due to the formation of atherothrombosis, likely leading to slow gait speed and cognitive impairment via vascular ischemic brain lesions [31, 32]. Besides that, elevated serum levels of pro-inflammatory markers such as tumor necrosis factor-alpha (TNF-α), interleukin-1β (IL-1β) and C-reactive protein (CRP) have been reported among smokers, contributing to muscle mass reduction and weakness [33, 34]. However, no associations were found between muscle mass and weakness with MCR incidence in this study, as these factors could be the consequences or symptoms of MCR [35]. Prior work has demonstrated that older adults who smoke exhibit greater cognitive deficits than non-smokers [36, 37], and it has been shown that the accumulation of oxidative stress due to cigarette smoking damages cells in the blood vessels, narrowing the arteries and reducing cerebral blood flow, leading to cognitive impairment [38]. Thus, our findings— not unexpectedly— indicate that smoking cessation among older adults has a protective role against the development of MCR syndrome and adverse health outcomes. Further investigation is needed to elucidate the underlying mechanisms linking smoking to MCR and to reconcile any inconsistencies with previous findings.

Prior work has also provided evidence indicating that smoking is associated with hypertension, where both factors contributed to an increased risk of cognitive and gait impairment through cerebrovascular pathology [11, 39]. In this study, hypertension was one of the predictors of MCR among older adults, where older adults with hypertension increased the risk of MCR by 62.9%. This finding is supported by a previous longitudinal study which indicates that high blood pressure accelerates gait slowing in well-functioning older adults [40]. Worsening gait and functional performance increased white matter hyperintensities among hypertensive individuals [41]. The harmful effect of high blood pressure on the brain could be due to cerebral vascularisation alterations, such as hypoperfusion, ischemic and hemorrhagic stroke, and white matter damage [11]. A previous study reported that the white matter pathology is strongly associated with impaired processing speed, leading to mild cognitive impairment and Alzheimer’s disease [42]. In accordance, several studies also have revealed that hypertension is one of the major contributors to the impairment of global and specific cognitive domains [43, 44]. Therefore, by implementing cognitive rehabilitation programs that specifically target the cognitive deficits associated with hypertension, older adults can potentially experience improvements in cognitive function, maintain or regain functional independence, and enhance their overall quality of life.

Poor verbal memory, as indicated by low performance in the RAVLT test, was a predictor of MCR among Malaysian older adults. Our finding is consistent with a previous cross-sectional study reporting that verbal fluency involving memory, executive, and processing speed components was associated with accelerated gait speed decline [45]. However, the association of verbal memory with longitudinal gait decline is still limited, and our findings suggest a potential value for a general view of cognitive function, not restricted to executive function and attention domain, in identifying older adults at greatest risk of mobility decline. On the other hand, Gifford et al. [46] reported that a subjective memory complaint, one of the MCR criteria, was related to aspects of verbal episodic memory, particularly lower immediate and delayed recall on a serial list-learning task. The objective episodic learning and memory impairments precede structural imaging evidence of hippocampal atrophy, reflecting that rapid forgetting is an early clinical marker of Alzheimer’s disease pathogenesis [47]. Therefore, this finding suggests that poor verbal memory could be an early indicator in detecting older adults with a high risk of MCR. Cognitive training programs focusing on memory enhancement techniques, mnemonic strategies, and memory exercises may help mitigate the progression of cognitive decline and reduce the risk of mobility impairment associated with MCR.

We also observed that poor functional status of older adults, as indicated by the lower score of the IADL scale, was a predictor of MCR. Accordingly, older adults who had a decline in IADL performance capacity has been linked with low gait speed and instability of the walking pattern [48, 49]. It is likely that the inactivity and limited functional capabilities contribute to the insidious loss of bone and muscle strength; thus, walking becomes less frequent and slower [49]. Moreover, slight difficulties in performing IADL are consistent with subjective cognitive decline [50]. Potentially, the onset of functional impairment in predicting subjective cognitive decline marks a transition to mild cognitive impairment and dementia [51]. In fact, the presence of IADL impairment not due to a concurrent physical condition seems to be a valid marker of prodromal Alzheimer’s disease [52]. Therefore, recognising impairment in IADL could contribute to the early identification of individuals with and beyond MCR. Integrating functional assessments into routine clinical evaluations may facilitate the early detection of cognitive impairment and prompt further diagnostic evaluation, leading to timely intervention and support for older adults at risk of dementia.

To the best of the author’s knowledge, this is the first study to propose simple combined MCR assessment tools consisting of all the significant predictors, such as smoking and hypertension status, performance in the RAVLT test and functional status as assessed using the IADL questionnaire. One of the strengths of this model is that it included multifactorial MCR risk with modifiable and nonmodifiable biological behavioural MCR risk factors. A recent study by Ayers et al. 2022 also developed a subjective MCR (MCR-S) screening assessment for discriminating MCR individuals and predicting the risk of dementia [53] with excellent specificity and sensitivity. MCR-S is defined using only cognitive and motoric complaints, whilst our model is a cumulative predictor from our longitudinal study, which is clinically more beneficial to identify those with a high risk of developing MCR, especially in the multi-ethnic population. Without an intention to replace the current objective MCR tool, MCR-S and our model are considered a more straightforward assessment to aid in detecting potential MCR cases, particularly in resource-poor settings. Thus, validating both models in external cohorts is recommended to identify the robustness in detecting people with a high risk of MCR.

The pooled prevalence of MCR syndrome from a multi-country study was 9.7% among older adults aged 60 years and above [4]. However, this is the first study to report the incidence of MCR in multi-ethnic Asian older adults aged 60 years above through a prospective cohort study with the inclusion of comprehensive and multiple predictors. The MCR incidence rate was 4.0 per 100 person-years in older adults without MCR syndrome at the baseline. The figure reported was lower than the incidence rate of cognitive frailty disclosed by a previous LRGS-TUA study with 7.1 per 100 person-years [27]. The reasons could be that this studied population was older, and most of them had developed severe geriatric syndromes, particularly frailty, cognitive impairment and even dementia, thus contributing to a lower incidence rate of MCR, an earlier stage of pre-dementia. It is also important to note that this study only reported an outcome after 3.5 years, while the previous one had an outcome after a five-year follow-up [27]. Thus, there is a need to establish longer years of follow-up with younger older adults to estimate the trend of the MCR incidence rate among older adults.

There are several strengths in this study. This is the first report on the incidence rate and predictors of MCR, particularly among multi-ethnic older adults in Malaysia, using longitudinal data. It should be noted that the health outcomes of older adults differ according to ethnicity and socioeconomic status; thus, the findings could be a stepping stone to designing specific interventions targeting older adults within multi-ethnic settings. Moreover, this study involved a wide range of parameters with detailed protocol covering several domains, including modifiable lifestyle-related factors (smoking and alcohol consumption), anthropometric measurements, body composition, cognitive function, physical performance, psychosocial status, and nutrient intake related to the development of MCR. However, a few limitations in the present study should be acknowledged when interpreting the findings. The medical history, IADL, and psychosocial function were assessed by means of self-reporting, which may result in over or under-estimation of prevalence. There may be bias from the self-reporting method. In addition, the high drop-out rate of participants during follow-up could contribute to under-representing the study population and underestimation of the MCR incidence rate in our study. The discrepancies of the participants’ characteristics who dropped out differed systematically from those remained in this study may potentially limiting the applicability of the findings to broader population. Therefore, it is suggested that future longitudinal studies should be conducted with a larger sample size, explore strategies to minimise dropout by addressing barriers to participation and account for younger older adults to reduce the drop-out rate.

## Conclusion

In conclusion, the incidence rate of MCR among multi-ethnic older adults in Malaysia was 4.0 per 100 person-years for those without MCR at baseline. Older adults who are smoking, had hypertension, poor verbal memory as assessed by the RAVLT test, and low functional status as indicated by low IADL score were significant predictors of MCR development. It should be noted that age, years of education, body fat percentage, and nutritional factors do not have an association with MCR incidence in this study. These findings can be used as guidelines for early identification and strategising preventive management of MCR to slow the rate or avoid adverse health outcomes among older populations. The predictors reported in this study offer preliminary support for intervention studies in the future that will work towards optimising the physical and cognitive health of older adults.

## Data Availability

The datasets generated and/or analysed during the current study are not publicly available to protect the confidentiality and anonymity of study participants but are available from the corresponding author at reasonable request.
